# Purse string loop assistance for intracorporeal stapled anastomosis during laparoscopic anterior resection

**DOI:** 10.1308/003588412X13171221591259k

**Published:** 2012-05

**Authors:** F Adaba, L Humphreys, RJ Longman

**Affiliations:** University Hospitals Bristol NHS Foundation TrustUK

## BACKGROUND

Delivering the anvil of a circular stapling device into the pelvis during a laparoscopic anterior resection can be technically challenging. Once the anvil has been secured into the lumen of the proximal colon with a purse string suture, only the stem of the anvil can be used to manipulate it into the pelvis and the head of the stapling device. We report a simple and effective technique that assists in this task.

## TECHNIQUE

**Figure 1:**
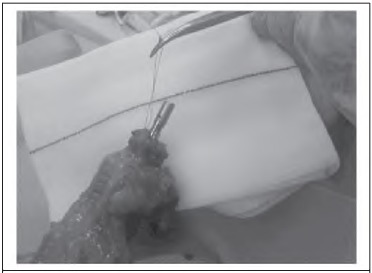
Loop suture on purse string

**Figure 2:**
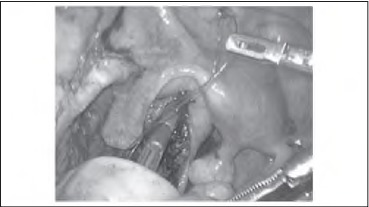
Laparoscopic grasper holding loop suture

The anvil is inserted into the proximal colonic lumen and secured with a purse string in the standard manner. After the knot has been tied on the purse string, a further loop is made on the suture and tied with a second knot (Fig 1). This loop can then be held with a laparoscopic grasper to assist with the manipulation of the anvil into the pelvis and subsequently to secure the anvil in the head of the stapling device (Fig 2). The suture loop is cut free on firing of the staple gun and can be withdrawn through a port.

## DISCUSSION

This cost neutral and effective technique can be used to simplify the process of docking the anvil of the circular stapling device with the head of the gun during a laparoscopic left-sided colonic resection. It is particularly useful in a narrow pelvis.

